# *Trichoderma* from Brazilian garlic and onion crop soils and description of two new species: *Trichoderma azevedoi* and *Trichoderma peberdyi*

**DOI:** 10.1371/journal.pone.0228485

**Published:** 2020-03-04

**Authors:** Peter W. Inglis, Sueli C. M. Mello, Irene Martins, João B. T. Silva, Kamilla Macêdo, Daniel N. Sifuentes, M. Cleria Valadares-Inglis

**Affiliations:** Embrapa Recursos Genéticos e Biotecnologia, Brasília, Brazil; Universidade de Coimbra, PORTUGAL

## Abstract

Fifty four *Trichoderma* strains were isolated from soil samples collected from garlic and onion crops in eight different sites in Brazil and were identified using phylogenetic analysis based on combined ITS region, *tef1-α*, *cal*, *act* and *rpb2* sequences. The genetic variability of the recovered *Trichoderma* species was analysed by AFLP and their phenotypic variability determined using MALDI-TOF. The strain clusters from both typing techniques coincided with the taxonomic determinations made from phylogenetic analysis. The phylogenetic analysis showed the occurrence of *Trichoderma asperellum*, *Trichoderma asperelloides*, *Trichoderma afroharzianum*, *Trichoderma hamatum*, *Trichoderma lentiforme*, *Trichoderma koningiopsis*, *Trichoderma longibrachiatum* and *Trichoderma erinaceum*, in the soil samples. We also identified and describe two new *Trichoderma* species, both in the harzianum clade of section *Pachybasium*, which we have named *Trichoderma azevedoi* sp. nov. and *Trichoderma peberdyi* sp. nov. The examined strains of both *T*. *azevedoi* (three strains) and *T*. *peberdyi* (12 strains) display significant genotypic and phenotypic variability, but form monophyletic clades with strong bootstrap and posterior probability support and are morphologically distinct from their respective most closely related species.

## Introduction

One of the most important fungal diseases occurring in garlic (*Allium sativum*) and onion (*Allium cepa*) is white rot, caused by the sclerotium-forming fungus *Sclerotium cepivorum*, often causing severe losses in garlic and onion production worldwide [1]. In Brazil, the states of Paraná, Minas Gerais, São Paulo and Goiás produce 64% of the national *Allium* crop (mostly garlic and onion) [2]. Despite recent advances in *Allium* production in Brazil, production is not sufficient to fulfil internal demand, due to low productivity [3]. Despite the diversity of garlic and onion cultivars available to growers, the favorable humidity and temperature conditions for most of these cultivars are also conducive to white rot disease. In the absence of reliable conventional white rot control methods, biological control is being investigated as a viable option, particularly using species of the fungal antagonist, *Trichoderma* [4].

*Trichoderma* has been widely used in biological control due to its ecological plasticity, easy large-scale production and efficiency against many plant pathogens such as *Fusarium*, *Pythium*, *Rhizoctonia*, *Sclerotinia*, *Botrytis* and *Verticillium* [5–9]. *Trichoderma* species are common in rhizospheric and non-rhizospheric soils and in endophytic relationships with many plants, displaying antifungal properties as well as promoting growth and inducing plant resistance against pathogenic fungi [10–12]. Three *Trichoderma asperellum* strains, one *Trichoderma harzianum* strain and a fifth unidentified *Trichoderma* strain from the rhizosphere of garlic and onion crops in Costa Rica have been tested for their *in vitro* antagonism against *S*. *cepivorum*, following their identification using ITS sequences [13]. The combination of different biocontrol agents to obtain synergistic or additive effects has also been tested in the field to control *S*. *cepivorum*, where simultaneous application of four selected species of *Trichoderma* (*Trichoderma hamatum*, *T*. *harzianum*, *Trichoderma oblongisporum* and *Trichoderma viride*), in association with fungicides, was shown to be effective for the management of white rot disease [14].

The phylogenetic species concept, based on concordance of multiple gene genealogies, has revolutionized fungal taxonomy [15] and exposed weaknesses in traditional morphology-based identification. Taxonomic revisions and the recognition of previously cryptic speciation in *Trichoderma* has also made clear that the universal DNA barcode for fungi, the internal transcribed spacers 1 and 2 of the nuclear ribosomal RNA gene cluster (ITS), is no longer adequate to ensure accurate species determinations in many *Trichoderma* sections [16–18], where a multi-gene approach is now usually adopted. By 2015 [19], there were 256 accepted *Trichoderma* name combinations, a number that is regularly increasing.

Multi-gene phylogenetics provides a gold standard for fungal identification and species delimitation. However, its methodology is time-consuming, technically demanding and expensive. Phenotyping using matrix-assisted laser desorption/ionization time-of-flight mass spectrometry (MALDI-TOF MS) provides an attractive alternative for rapid microbial identification and strain differentiation purposes and has been used in filamentous fungi such as species of *Aspergillus*, *Fusarium*, *Penicillium*, *Trichoderma* and *Metarhizium*, among others [20,21]. The major advantages of MALDI-TOF are its cost effectiveness, rapidity, low error rate and the possibility of distinguishing closely related species [22].

While correct species identification is important in the selection and validation of microbial biocontrol agents [23], assessment of infraspecific variation is also of importance to protect commercial strains and to understand the genetic resources available in natural populations. There are abundant reports of molecular genotyping techniques applied to fungal biocontrol agents available in the literature. One of the most attractive methods, however, due to its ability to efficiently generate large numbers of markers at low cost which are amenable to automated fluorescence-based scoring is AFLP (amplification fragment length polymorphism), which has been used to identify and differentiate closely related species of *Trichoderma* [24].

Due to the potential of *Trichoderma* species to control white rot disease, we aimed to collect strains from crop soils from multiple localities in some of the principal garlic and onion growing areas in Brazil. We also aimed to correctly identify the strains, under the current taxonomic framework, to the species level using multi-gene DNA sequence analysis and assess their genetic and phenotypic variation using AFLP and MALDI-TOF, respectively. Such data will be a valuable resource for ongoing biocontrol research in Brazil.

## Materials and methods

### Collection and isolation of *Trichoderma* strains

**Fig 1:**
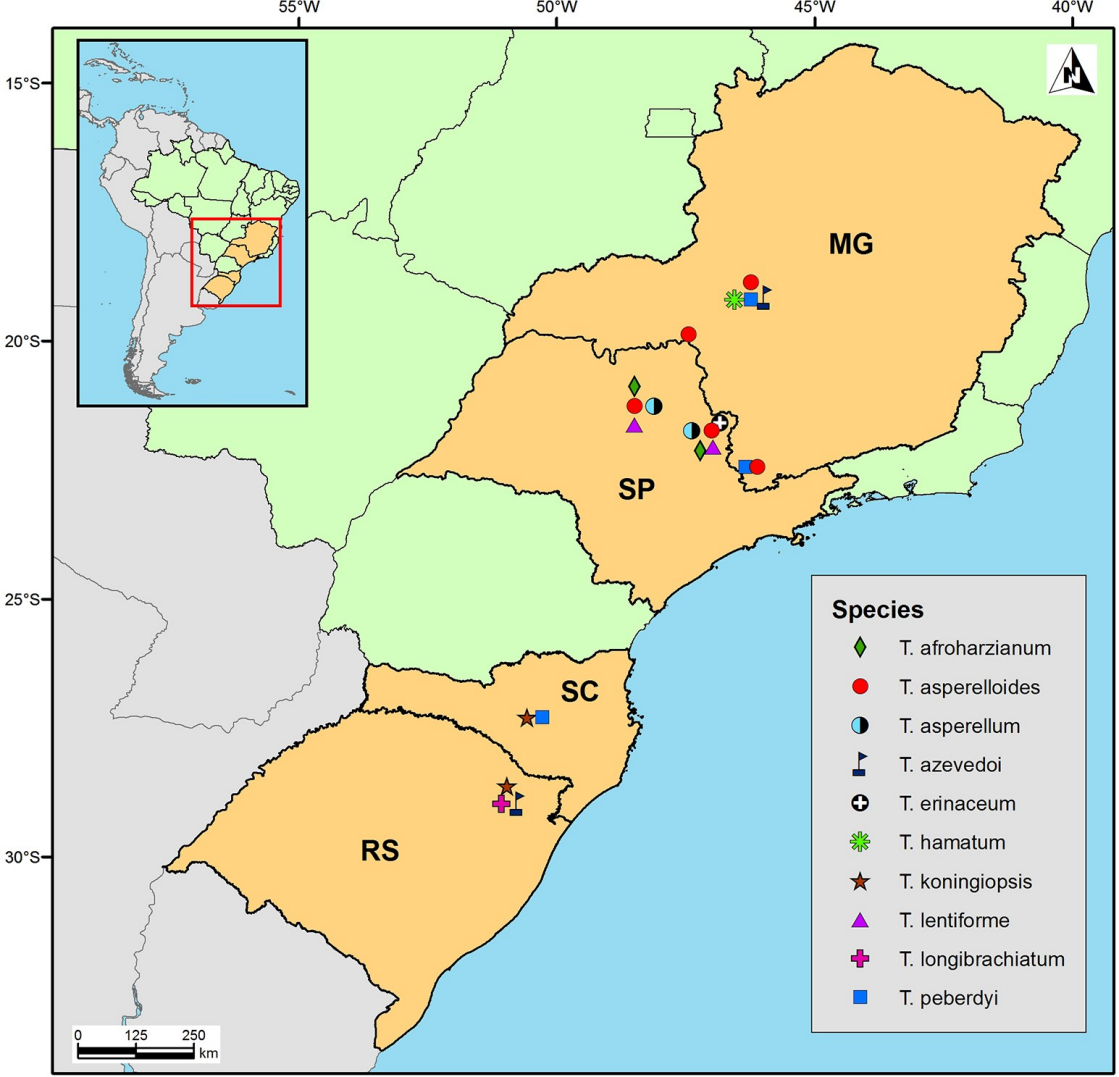
Map of Southeastern Brazil showing soil collection sites and recovered *Trichoderma* species.

*Trichoderma* strains were isolated from soil samples collected from eight distinct garlic or onion crops in the Brazilian states of Santa Catarina (SC), Minas Gerais (MG), Rio Grande do Sul (RS) and São Paulo (SP) (Table 1; Fig 1 - Map). From each sample, 10 g of soil was placed in a 250 ml Erlenmeyer flask containing 90 ml of sterile distilled water. After stirring at 180 rpm for 40 min, serial dilutions were spread onto plates containing Martin's semi-selective medium (per litre: 18 g agar, 10 g dextrose, 0.5 g MgSO_4_, 0.5 g peptone, 0.5 g beef extract, 0.05 g bengal pink and 0.3 g chloramphenicol) and incubated at 28°C for 7 days. Isolated colonies with typical *Trichoderma* morphology were transferred to potato-dextrose agar (PDA; Difco) supplemented with 0.25 ml l^-1^ Triton X100 and 0.3 g l^-1^ chloramphenicol for the subsequent isolation of monosporic cultures. Fungal sample collection was carried out according to Brazilian legislation (IBAMA process 02001.006479/2010-93 and permit n^o^ 02/2008).

**Table 1:** *Trichoderma* species identified from garlic and onion crop soils in Brazil and Genbank accession numbers of their partial actin, calmodulin, *rpb2*, *tef1-α* and ITS sequences used in phylogenetic analysis.

Species	Strain	Collection location	Crop	*act*	*cal*	*rpb2*	*tef1-α*	ITS
*T*. *koningiopsis*	CEN1386	Curitibanos, SC	Garlic	MK696725	MK696671	MK696779	MK696617	MK714859
*T*. *peberdyi*	CEN1387	Curitibanos, SC	Garlic	MK696727	MK696673	MK696781	MK696619	MK714861
*T*. *peberdyi*	CEN1388	Curitibanos, SC	Garlic	MK696728	MK696674	MK696782	MK696620	MK714862
*T*. *peberdyi*	CEN1389	Curitibanos, SC	Garlic	MK696729	MK696675	MK696783	MK696621	MK714863
*T*. *peberdyi*	CEN1390	Curitibanos, SC	Garlic	MK696730	MK696676	MK696784	MK696622	MK714864
*T*. *peberdyi*	CEN1391	Rio Paranaíba, MG	Garlic	MK696731	MK696677	MK696785	MK696623	MK714865
*T*. *peberdyi*	CEN1392	Rio Paranaíba, MG	Garlic	MK696732	MK696678	MK696786	MK696624	MK714866
*T*. *peberdyi*	CEN1393	Rio Paranaíba, MG	Garlic	MK696734	MK696680	MK696788	MK696626	MK714868
*T*. *hamatum*	CEN1394	Rio Paranaíba, MG	Garlic	MK696735	MK696681	MK696789	MK696627	MK714869
*T*. *hamatum*	CEN1395	Rio Paranaíba, MG	Garlic	MK696736	MK696682	MK696790	MK696628	MK714870
*T*. *asperelloides*	CEN1396	Rio Paranaíba, MG	Garlic	MK696737	MK696683	MK696791	MK696629	MK714871
*T*. *asperelloides*	CEN1397	Rio Paranaíba, MG	Garlic	MK696738	MK696684	MK696792	MK696630	MK714872
*T*. *peberdyi*	CEN1398	Bueno Brandão, MG	Garlic	MK696740	MK696686	MK696794	MK696632	MK714874
*T*. *longibrachiatum*	CEN1399	São Marcos, RS	Garlic	MK696741	MK696687	MK696795	MK696633	MK714875
*T*. *longibrachiatum*	CEN1400	São Marcos, RS	Garlic	MK696742	MK696688	MK696796	MK696634	MK714876
*T*. *longibrachiatum*	CEN1401	São Marcos, RS	Garlic	MK696744	MK696690	MK696798	MK696636	MK714878
*T*. *longibrachiatum*	CEN1402	São Marcos, RS	Garlic	MK696745	MK696691	MK696799	MK696637	MK714879
*T*. *azevedoi*	CEN1403	São Marcos, RS	Garlic	MK696746	MK696692	MK696800	MK696638	MK714880
*T*. *longibrachiatum*	CEN1404	São Marcos, RS	Garlic	MK696747	MK696693	MK696801	MK696639	MK714881
*T*. *koningiopsis*	CEN1405	São Marcos, RS	Garlic	MK696749	MK696695	MK696803	MK696641	MK714883
*T*. *koningiopsis*	CEN1406	São Marcos, RS	Garlic	MK696750	MK696696	MK696804	MK696642	MK714884
*T*. *koningiopsis*	CEN1407	São Marcos, RS	Garlic	MK696751	MK696697	MK696805	MK696643	MK714885
*T*. *asperelloides*	CEN1408	Monte Alto, SP	Onion	MK696753	MK696699	MK696807	MK696645	MK714887
*T*. *asperelloides*	CEN1409	Monte Alto, SP	Onion	MK696755	MK696701	MK696808	MK696647	MK714889
*T*. *afroharzianum*	CEN1410	Monte Alto, SP	Onion	MK696756	MK696702	MK696809	MK696648	MK714890
*T*. *asperelloides*	CEN1411	Monte Alto, SP	Onion	MK696757	MK696703	MK696810	MK696649	MK714891
*T*. *lentiforme*	CEN1412	Monte Alto, SP	Onion	MK696758	MK696704	MK696811	MK696650	MK714892
*T*. *asperelloides*	CEN1413	Monte Alto, SP	Onion	MK696759	MK696705	MK696812	MK696651	MK714893
*T*. *afroharzianum*	CEN1414	Monte Alto, SP	Onion	MK696760	MK696706	MK696813	MK696652	MK714894
*T*. *lentiforme*	CEN1415	São José do Rio Pardo, SP	Onion	MK696761	MK696707	MK696814	MK696653	MK714895
*T*. *lentiforme*	CEN1416	São José do Rio Pardo, SP	Onion	MK696762	MK696708	MK696815	MK696654	MK714896
*T*. *afroharzianum*	CEN1417	São José do Rio Pardo, SP	Onion	MK696763	MK696709	MK696816	MK696655	MK714897
*T*. *asperelloides*	CEN1418	São José do Rio Pardo, SP	Onion	MK696764	MK696710	MK696817	MK696656	MK714898
*T*. *asperelloides*	CEN1419	São José do Rio Pardo, SP	Onion	MK696765	MK696711	MK696818	MK696657	MK714899
*T*. *erinaceum*	CEN1420	São José do Rio Pardo, SP	Onion	MK696766	MK696712	MK696819	MK696658	MK714900
*T*. *erinaceum*	CEN1421	São José do Rio Pardo, SP	Onion	MK696767	MK696713	MK696820	MK696659	MK714901
*T*. *azevedoi*	CEN1422	Rio Paranaíba, MG	Onion	MK696768	MK696714	MK696821	MK696660	MK714902
*T*. *azevedoi*	CEN1423	Rio Paranaíba, MG	Onion	MK696769	MK696715	MK696822	MK696661	MK714903
*T*. *asperelloides*	CEN1424	Rio Paranaíba, MG	Onion	MK696770	MK696716	MK696823	MK696662	MK714904
*T*. *peberdyi*	CEN1425	Rio Paranaíba, MG	Onion	MK696771	MK696717	MK696824	MK696663	MK714905
*T*. *peberdyi*	CEN1426	Itobi, SP	Onion	MK696772	MK696718	MK696825	MK696664	MK714906
*T*. *asperelloides*	CEN1427	Itobi, SP	Onion	MK696773	MK696719	MK696826	MK696665	MK714907
*T*. *lentiforme*	CEN1428	Itobi, SP	Onion	MK696775	MK696721	MK696827	MK696667	MK714909
*T*. *lentiforme*	CEN1429	São José do Rio Pardo, SP	Onion	MK696776	MK696722	MK696828	MK696668	MK714910
*T*. *asperelloides*	CEN1430	São José do Rio Pardo, SP	Onion	MK696777	MK696723	MK696829	MK696669	MK714911
*T*. *asperelloides*	CEN1431	Sacramento, MG	Onion	MK696778	MK696724	MK696830	MK696670	MK714912
*T*. *peberdyi*	CEN1457	Curitibanos, SC	Garlic	MK696726	MK696672	MK696780	MK696618	MK714860
*T*. *peberdyi*	CEN1458	Curitibanos, SC	Garlic	MK696733	MK696679	MK696787	MK696625	MK714867
*T*. *asperelloides*	CEN1459	Bueno Brandão, MG	Garlic	MK696739	MK696685	MK696793	MK696631	MK714873
*T*. *longibrachiatum*	CEN1460	São Marcos, RS	Garlic	MK696743	MK696689	MK696797	MK696635	MK714877
*T*. *longibrachiatum*	CEN1461	São Marcos, RS	Garlic	MK696748	MK696694	MK696802	MK696640	MK714882
*T*. *longibrachiatum*	CEN1462	São Marcos, RS	Garlic	MK696752	MK696698	MK696806	MK696644	MK714886
*T*. *asperellum*	CEN1463	Monte Alto, SP	Onion	MK696754	MK696700	-	MK696646	MK714888
*T*. *asperellum*	CEN1464	Itobi, SP	Onion	MK696774	MK696720	-	MK696666	MK714908

### Morphological characterization

For comparison of growth, colony appearance and morphological features, discs of fresh monosporic *Trichoderma* cultures were transferred to 9 cm Petri dishes containing 20 ml of either PDA, CMD (cornmeal dextrose agar) or SNA (synthetic low nutrient agar), which were cultured at 15, 20, 25, 30 and 35°C, with 12 hour photoperiod. Morphological characteristics, such as the aspects of phialides, conidia and chlamydospores were observed using a Nikon Eclipse Ci microscope fitted with a Nikon DS Ri2 camera. Microscopical measurements and analysis were carried out using NIS Elements (v. 4.30.01, Nikon) software, where means were based on 30 individual phialides and conidia from each specimen.

### Phylogenetic analysis

Strains were cultivated on PDA for 72 h at 25°C prior to collection of mycelium, which was scraped from the agar surface, lyophilized and maintained at -80°C. Genomic DNA was purified from approximately 20 mg of the lyophilized mycelium, using a cetyl trimethyl ammonium bromide (CTAB) extraction method [25]. The nuclear ribosomal ITS1–5.8S rRNA–ITS2 region (ITS), actin (*act*), calmodulin (*cal*), translation elongation factor 1-α (*tef1-α*) and RNA Polymerase II subunit (*rpb2*) markers were amplified by PCR using a mix comprising approximately 2 ng genomic DNA, 1x PCR buffer with 2.0 mM MgCl_2_, 0.2 mM dNTPs, 1U *Taq* polymerase and 0.3 μM of each primer. Thermal cycling for all markers was standardized as 2 min at 95°C then 35 cycles of 20 sec at 95°C, 30 sec at the appropriate annealing temperature for the primers used and 90 sec at 72°C, followed by 7 min at 72°C. Primer sequences and annealing temperatures are given in Table 2. The internal *act* sequencing primer, Tact293F, was designed from a conserved region identified in a preliminary alignment of several *Trichoderma act* PCR products sequenced using the amplification primers, along with cognate reference sequences obtained from Genbank. The *tef1-α* reverse PCR primer, tef1080R, was designed from a conserved region identified in an alignment of the 3´ portion of the *tef1-α* gene, amplified from a selection of *Trichoderma* isolates using EF1–1018F and EF1–1620R primers. We obtained fewer artefact bands in PCRs using the tef1080R reverse primer compared with the more widely used tef997R primer. Since the 3´ portion of the *tef1-α* gene is much less variable than the 5´ portion, phylogenetic analysis was restricted to the portion amplified using tef71f and tef1080R primers. Furthermore, there is a richer representation of the 5´ portion of the *tef1-α* gene from related *Trichoderma* species in the databanks, further influencing our decision.

**Table 2:** Primers used for PCR and sequencing.

Locus	Name	Primer sequence 5´-3´	T_m_	Reference
ITS	U1	GGAAGKARAAGTCGTAACAAGG	55	[29]
	U4	RGTTTCTTTTCCTCCGCTTA		"
*act*	Tact1	TGGCACCACACCTTCTACAATGA	50	[30]
	Tact2	TCTCCTTCTGCATACGGTCGGA		[31]
*	Tact511R	CTCAGGAGCACGGAAT		"
*	Tact293F	GTGATCTTACCGACTACCTGATG		This study
*cal*	CAL-228F	GAGTTCAAGGAGGCCTTCTCCC	55	[32]
	CAL-737R	CATCTTTCTGGCCATCATGG		"
*	CAL-235F	TTCAAGGAGGCCTTCTCCCTCTT		"
*tef1-α*	tef71f	CAAAATGGGTAAGGAGGASAAGAC	50	[33]
*	tef85f	AGGACAAGACTCACATC AACG		"
*	tef954r	AGTACCAGTGATCATGTTCTTG		"
*	tef997R	CAGTACCGGCRGCRATRATSAG		"
	tef1080R	GATACCAGCCTCGAACTCACC		This study
	EF1–1018F	GAYTTCATCAAGAACATGAT		[34]
	EF1–1620R	GACGTTGAADCCRACRTTGTC		"
*rpb2*	RPB2_210up	TGGGGWGAYCARAARAAGG	48	Tom Gräfenhan;
	RPB2_1450low	CATRATGACSGAATCTTCCTGGT		http://www.isth.info
*	RPB2 1150low	GGTTGTGATCRGGRAARGGAATG		"

*Internal primers used for sequencing only.

PCR products were verified by agarose gel electrophoresis and were then prepared for sequencing using ExoSAP (Applied Biosystems, Foster City, CA, USA). Both DNA strands were sequenced using the Big Dye v.3.1 kit (Applied Biosystems), using appropriate primers (Table 2) and an ABI3730 DNA Analyzer (Applied Biosystems). Sequence reads were trimmed for quality, contigs assembled and any base calling mismatches resolved using Chromas Pro (v. 1.5, Technelysium Pty Ltd). Sequences, including references obtained from Genbank, were organized into matrices in Bioedit (v. 7.2.6) [26] and aligned using MAFFT v. 7 E-INS-i [27]. A concatenated matrix was assembled using Sequence Matrix (v. 1.8) [28].

An optimal partitioning scheme for each marker was determined in PartitionFinder 2 [35]. Maximum likelihood trees based on data from each marker as well as the concatenated matrix were constructed using IQ-TREE (v. 1.6.5) [36], where optimal nucleotide substitution models for each partition were selected using ModelFinder [37]. Branch support was estimated using the Ultrafast bootstrap (UFBoot) [38] with 1000 replicates. Branches with UFboot support of > = 95% were considered credible. Representative sequences of each *Trichoderma* isolate cluster for each marker were used in BLAST [39] searches of the Genbank database in order to make provisional *Trichoderma* species identifications. This information was used to select reference sequences for further analyses, also incorporating strains used in recent taxonomic and molecular phylogenetic treatments of appropriate *Trichoderma* sections [23,40,41], S1 Table.

The partitioned concatenated matrix was also analysed using the Bayesian Metropolis-coupled Markov Chain Monte Carlo method as implemented in MrBayes 3.2.6 [42], running on the CIPRES Science Gateway [43] and utilizing the Beagle library [44]. Model selection for each partition was made in PartitionFinder2 [35]. Two runs of eight MCMCMC chains with a heating temperature of 0.075 were conducted for ten million generations, sampling every 1000 generations. This runtime was sufficient for the convergence diagnostic, the standard deviation of split frequencies, to fall to a minimum of 0.005217. The first 25% of the trees were discarded (burn-in) prior to calculation of the 50% majority rule consensus tree.

### AFLP genotyping

Genetic variability among a representative selection of 46 of the 54 *Trichoderma* isolates was evaluated using the amplified fragment length polymorphism method (AFLP; [45]; adapter and primer sequences given in Table 3) adapted for fluorescent detection. A one-step digestion and adapter-ligation protocol was adopted, which were performed in 20 μl volumes. A single reaction comprised 1X ligase buffer (Promega), 50 mM NaCl, 0.05 μg/μl bovine serum albumin, one unit T4 DNA ligase (Promega), five pmol EcoRI adapter, 50 pmol *Mse*I adapter, five units *Eco*RI (*Eco*RI-HF high fidelity, NEB), five units of *Mse*I and 100 ng genomic DNA. The reactions were incubated on a PCR machine at 37°C for two hours, then held at 17°C for one hour and then held at 4°C for two hours. Samples were then diluted five times by the addition of 80 μl H_2_O and stored at -80°C. The primers *Eco*RI+A and *Mse*I+C were used for preselective PCR. A 20 μl PCR in 1X PCR Buffer with 2 mM Mg^2+^ contained 1 M Betaine, 0.25 mM dNTPs, 0.5 μM each primer, 1 U *Taq* polymerase and 2 μl of the diluted adapter-ligated DNA. Cycling conditions comprised an initial 72°C for 2 min to allow fill-in of the adapter ends, then 20 cycles of 94°C for 30 sec, 56°C for 1 min and 72°C for 2 min. Ramp rate was limited to 1°C per second. Following cycling, reactions were held at 72°C for 2 min and then 60°C for 30 min. Five μl of PCR products were subsequently analysed on a 1.5% agarose gel, producing a faint smear if the reaction was successful. The preselected DNA was then diluted five times by the addition of 80 μl H_2_O and stored at -20°C.

**Table 3:** AFLP adapter and primer sequences.

Primer name	Primer sequence 5´-3´
*Eco*RI-Adapter1	CTCGTAGACTGCGTACC
*Eco*RI-Adapter2	AATTGGTACGCAGTCTAC
*Eco*RI+A	GACTGCGTACCAATTCA
*Mse*I_Adapter1	GACGATGAGTCCTGAG
*Mse*I_Adapter2	TACTCAGGACTCAT
*Mse*I+C	GATGAGTCCTGAGTAAC
(11) *Eco*RI-AC FAM	FAM+GACTGCGTACCAATTCAC
(14) *Eco*RI-AA VIC	VIC+GACTGCGTACCAATTCAA
(2) *Mse*I+CTT	GATGAGTCCTGAGTAACTT
(3) *Mse*I+CAT	GATGAGTCCTGAGTAACAT
(5) *Mse*I+CAG	GATGAGTCCTGAGTAACAG
(6) *Mse*I+CAA	GATGAGTCCTGAGTAACAA

Genomic complexity was further reduced using selective PCR to produce resolvable AFLP profiles, using PCR primer pairs comprising one labelled *Eco*RI+2 and one unlabelled *Mse*I+3 primer (Table 3). Selective primer combinations producing an adequate number of clear fluorescent peaks in preliminary screening were chosen from the range available in the Small Plant Genome Mapping Kit (Applied Biosystems). We found that whilst most *Mse*I+3 primer variations gave satisfactory results, only the *Eco*RI+AC and *Eco*RI+AA primers were efficient in *Trichoderma*. A single 10 μl selective PCR reaction in 1X PCR buffer with 2 mM Mg^2+^ contained 0.15 μM each of *Mse*I+3 primer and fluorochrome-labelled *Eco*RI+2 primer, 0.2 mM dNTPs, 0.5 U *Taq* polymerase and 2 μl of diluted preselected DNA. PCR cycling comprised an initial 94°C for 2 min, then 10 cycles of 94°C for 30 sec, 66°C for 30 sec and 72°C for 1 min. The annealing temperature was reduced by 1°C per cycle (touchdown). Then followed 25 cycles of 94°C for 30 sec, 56°C for 30 sec and 72°C for 1 min. Reactions were then held at 72°C for 3 min and at 60°C for 30 min. Six primer combinations (11x5, 11x6, 14x2, 14x3, 14x5, 14x6; Table 3) were selected for the full analysis. The fluorescent AFLP profiles were detected by mixing 1 μl PCR product with 9 μl HiDi formamide and 0.3 μl of the Genescan 600-LIZ v 2.0 molecular size ladder (Applied Biosystems). Samples were denatured at 95°C for 5 minutes and snap cooled on ice, prior to injection on an ABI 3730 DNA Analyzer (Applied Biosystems).

The raw AFLP data files were processed using PeakScanner (v. 2; Applied Biosystems). The table of peak area data was then imported into the R CRAN library program, RawGeno [46], for peak binning and filtering of low quality or partially overlapping peaks, thereby reducing the risk of size-homoplasy. The filtered AFLP profiles were then converted into a peak presence or absence binary matrix, totalling 364 characters. Data was analysed under the F81 (restriction; nst = 1 rates = invgamma) model in MrBayes 3.2.6 [42] using two runs of four MCMCMC chains, where two million generations were sampled every 1000 generations. This runtime was sufficient for the average standard deviation of split frequencies to fall to 0.007734. The first 25% of the trees were discarded (burn-in) prior to calculation of the 50% majority rule consensus tree. Intraspecific Nei-Li genetic distances (fragments, length = 4) were calculated in PAUP (v. 4.0a165) [47].

### MALDI-TOF phenotyping and rapid identification

Samples of *Trichoderma* strains were collected from colonies cultivated on PDA plates, which were applied directly to a MSP96 plate (Bruker Daltonics GmbH, Bremen, Germany) and covered with 1 μl of MALDI matrix solution (‘Bruker HCCA’ or α-cyano-4-hydroxycinnamic acid, at a final concentration of 5 mg HCCA ml^-1^). After sample drying, analyses were performed on a MicroFlex MALDI-TOF mass spectrometer (Bruker Daltonics GmbH), fitted with a nitrogen laser (337 nm) of 20–65% offset intensity and spiral mode of acquisition, where an average of 400 shots (40 laser shots at 10 different regions of the target spot) at 60 Hz were conducted. Signals in the range 2000–20000 m/z were automatically collected with AutoConverter from the acquisition software (FlexControl 3.3; Bruker Daltonics GmbH). Data were exported to MALDI Biotyper software (3.0; Bruker Daltonics GmbH) and each consensus spectrum incorporated into a profile in the mean spectrum projection (MSP) database.

Based on the results of the phylogenetic analysis, the strains CEN1386, CEN1395, CEN1402, CEN1419, CEN1390, CEN1416 and CEN1420, were selected for the creation of a local *Trichoderma* spectrum database using the Biotyper MBT Explorer Software Module. The library was constructed by sampling colonies grown on individual PDA plates for five days, where material was collected from three distinct regions (colony edge, intermediate and central). The spectra obtained were then used to generate species profiles which were added to the database. Subsequent samples were analyzed in triplicate. Cluster analysis was conducted using the MSP Dendrogram Creation Standard Method (v.1.4) of MALDI Biotyper Software (v. 3.0).

### Nomenclature

The electronic version of this article in Portable Document Format (PDF) in a work with an ISSN or ISBN will represent a published work according to the International Code of Nomenclature for algae, fungi, and plants, and hence the new names contained in the electronic publication of a PLOS ONE article are effectively published under that Code from the electronic edition alone, so there is no longer any need to provide printed copies.

In addition, new names contained in this work have been submitted to MycoBank from where they will be made available to the Global Names Index. The unique MycoBank number can be resolved and the associated information viewed through any standard web browser by appending the MycoBank numbers contained in this publication to the prefix http://www.mycobank.org/MB/. The online version of this work is archived and available from the following digital repositories: PubMed Central, LOCKSS.

## Results and discussion

A total of 54 *Trichoderma* strains were isolated from crop soil samples from multiple sites representing the main growing areas of garlic and onion in Brazil. In quantitative terms, 11 strains were isolated from Rio Paranaíba, MG; one from Bueno Brandão, MG; one from Sacramento, MG; seven from Monte Alto, SP; nine from São José do Rio Pardo, SP; three from Itobi, SP; nine from São Marcos, RS; and five from Curitibanos, SC (Table 1; Fig 1).

All 54 presumptive strains were confirmed as *Trichoderma* species using the ITS oligonucleotide barcode identification program *Trich*OKEY2 [48] http://www.isth.info/. However, confident and unambiguous species identifications were not obtained in the searches, as expected, since previous studies have pointed out the limitations of ITS sequences to delimit *Trichoderma* species [23]. Furthermore, 12 isolates were returned as belonging to unidentified *Trichoderma* species. We therefore performed a full phylogenetic analysis on all 54 isolates with the addition of a further four phylogenetic markers: *act*, *cal*, *tef1-α* and *rpb2*. We first performed a ML phylogenetic analysis on the most highly substituted data set (*tef1-α*; Table 4). Clusters with high sequence similarity were identified and a representative sequence of each cluster used in BLAST [39] searches of the Genbank nucleotide database. Reference sequences for each marker were then selected from the *Trichoderma* (or its sexual morph, *Hypocrea*) species producing top hits, giving preference to type strains and those with most complete representation for our marker selection. We also selected reference sequences based on recent molecular taxonomic treatments of the *Trichoderma* sections and major clades identified in the initial ITS *Trich*OKEY screen (S1 Table).

**Table 4:** Partition statistics for each sequenced DNA locus generated in IQ-TREE.

Partition	Sites	Invariable sites	Parsimony informative sites	Tree length	Best fit model (BIC)
*act*	754	579	135	1.1454	TIM3e+I+G4
*cal*	524	216	263	3.2113	K2P+I+G4
ITS	685	405	144	1.8155	TIM2+F+R3
*rpb2*	800	471	295	2.7089	TIMe+I+G4
*tef1-α*	752	272	405	9.7299	TN+F+R4

Among the sequenced markers, the most informative, based on number of parsimony informative characters (PICS), was *tef1-α*, followed by *rpb2*, *cal*, ITS and *act* (Table 4). The *tef1-α* matrix, including reference sequences, was notably rich in indels, presenting a potential risk for alignment ambiguity, which we attempted to minimize by the use of MAFFT E-INS-i, which is among the most accurate of modern consistency-based programs [49]. Among the 54 isolates studied, sequence data were gathered for all five markers, except for two isolates, later identified as *Trichoderma asperellum*, for which the *rpb2* marker could not be amplified, probably due to critical primer mismatch.

**Fig 2:**
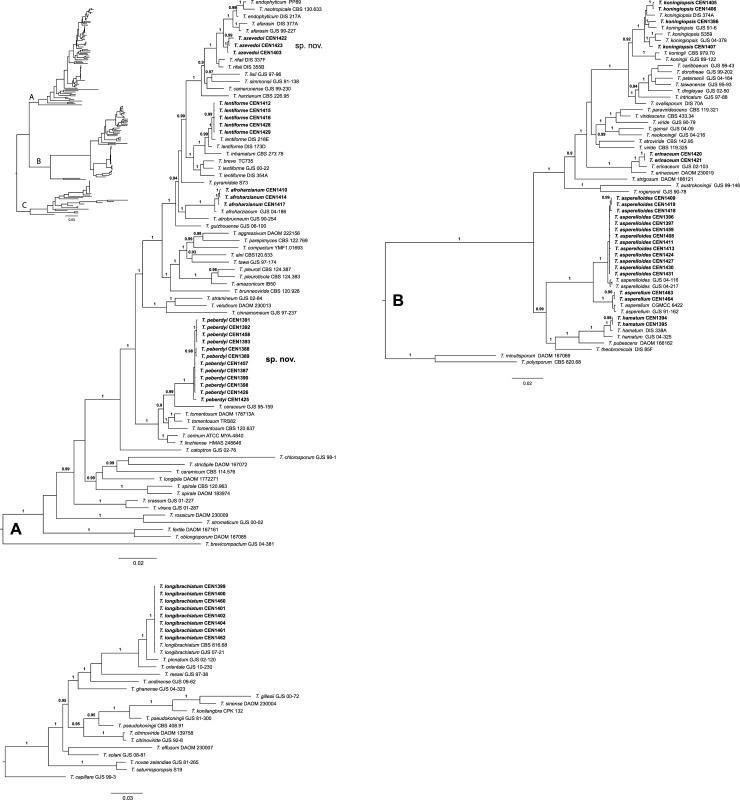
A-C. Midpoint rooted Bayesian phylogram (split into three parts as indicated in the overview), based on the concatenation of *act*, *cal*, ITS, *rpb2* and *tef1-α* matrices. Posterior probabilities are given above branches (> 0.9) and the scale bar represents expected changes per site. Strains sequenced in the present study are in bold and are followed by CENxxx numbers. Two new *Trichoderma* species, *T*. *azevedoi* and *T*. *peberdyi* are indicated.

The Bayesian phylogram based on the concatenation of ITS, *rpb2*, *act*, *cal* and *tef1-α* sequences (Fig 2) permitted the unambiguous identification of eight species among the 54 isolates, based on their clustering with reference taxa. The isolates identified in the analysis included five *Trichoderma lentiforme*, two *Trichoderma hamatum*, three *Trichoderma afroharzianum*, four *Trichoderma koningiopsis*, two *Trichoderma erinaceum*, 13 *Trichoderma asperelloides*, two *T*. *asperellum* and eight *Trichoderma longibrachiatum*. The remaining 15 isolates formed two distinct clades: one comprising three isolates, most closely related to *Trichoderma rifaii*, *Trichoderma afarasin*, *Trichoderma endophyticum* and *Trichoderma neotropicale* and a second clade comprising 12 isolates, corresponding to the unidentified group in the TrichOKEY search, most closely related to *Trichoderma ceraceum* and *Trichoderma tomentosum*. Both clades are highly supported in the Bayesian analysis (PP = 1) (Fig 2) and possess 100% ultrafast bootstrap support in a maximum likelihood tree based on the same concatenated matrix (S1 Fig). We therefore suspected that the two unidentified clades represent new *Trichoderma* species, which we confirmed by examination of their distinctive growth and morphological characteristics.

### Taxonomy

#### *Trichoderma azevedoi* Valadares-Inglis, M.C. & Inglis, P.W. sp. nov. Fig 3

**Fig 3:**
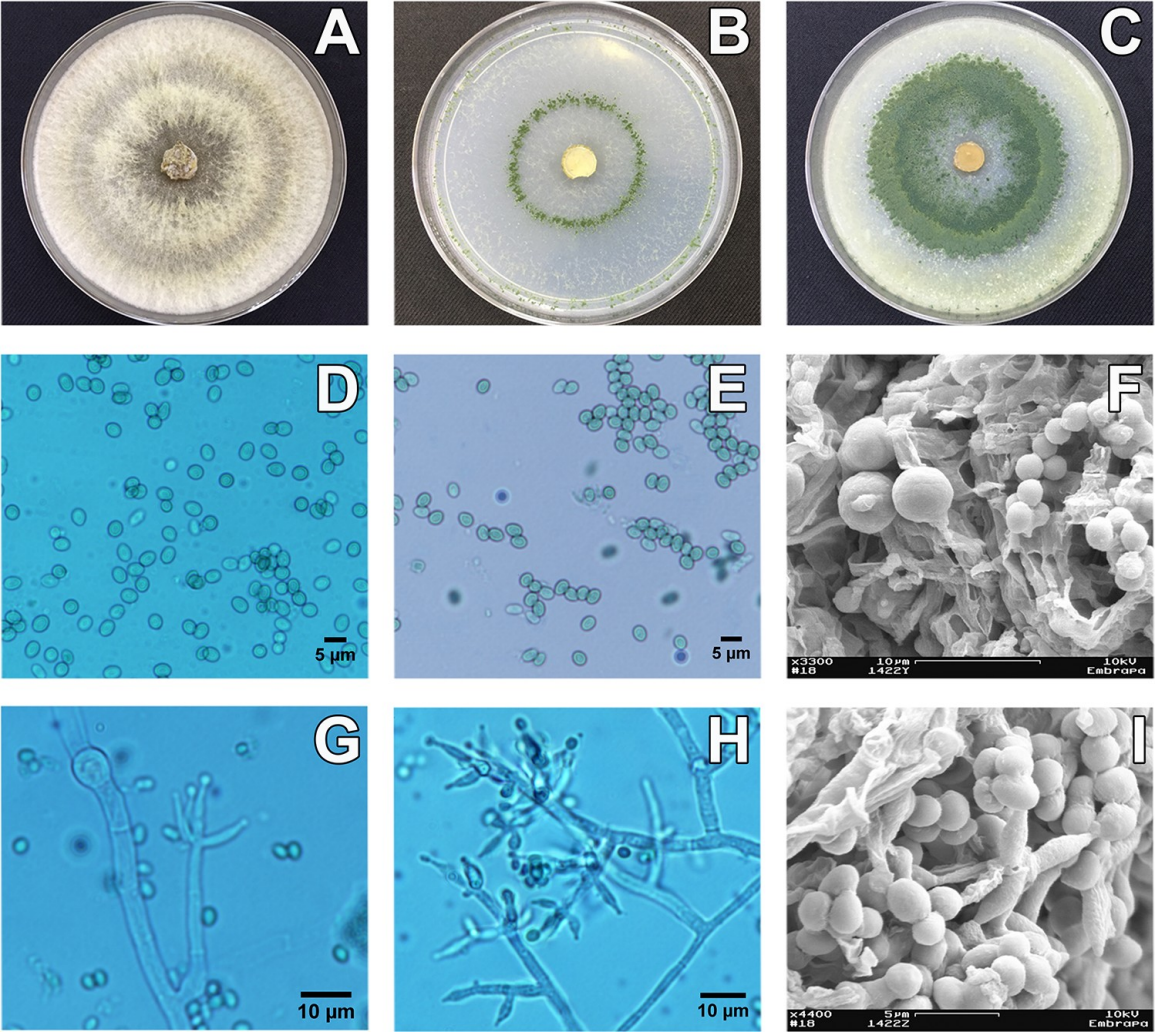
Culture characteristics and morphology of *T. azevedoi* sp. nov. strain CEN1422 (holotype). Panels A-I: Growth on three different media, PDA (A); SNA (B); CMD (C); and morphology of the conidia (D, E), phialides (G, H) and chlamydospores (G) using optical microscopy and conidia and chlamydospores using electron microscopy (F, I).

Mycobank MB830305. [urn:lsid:mycobank.org: 830305]

#### Etmology

Named in honour of João Lúcio Azevedo (São Paulo University—Brazil) for his contributions to mycology and microbial genetics in Brazil, including the mentoring of numerous professionals in *Trichoderma* studies.

#### Holotype

CEN1422, a freeze dried, metabolically inactive culture deposited in the Herbarium of Embrapa Recursos Genéticos e Biotecnologia (CEN). Collected in Rio Paranaíba—MG state, Brazil, 20^0^ 05^’^ 06” S, 51^0^ 00^’^ 02^”^ E, from onion crop soil, 02/07/2015, by V. Lourenço Jr. & J.B.T. da Silva. An ex-holotype culture of CEN1422 has been deposited in the Embrapa Coleção de Microrganismos para o Controle de Fitopatógenos e Plantas Daninhas, with the accession number BRM46357.

#### Description

On CMD, colony radius 40 mm after 72h at 25 and 30°C and 12 h photoperiod. Mycelium hyaline with cottony pustules, sporulating heavily after 72 h at 30°C and 96 h at 25°C, turning green after 96 h, more abundantly in a broad ring about half-way to the plate center. At 20°C and 12 h photoperiod, colony radius 25 mm, mycelium hyaline with pustules of spores formed at 96h. No growth observed at 15 and 30°C. On SNA, colony radius 34 mm at 25°C and 40 mm at 30°C after 72 h with 12 h photoperiod. Mycelium hyaline with spores formed after 72 h in sparse clumps distributed throughout the plate. At 30°C mid-green spores are formed in a distinct thin concentric ring, approximately one third of the radius from the plate center to the edge. At 20°C spores are produced after 96 h similarly to 30°C. On PDA, colony radius 17 mm at 20°C, 62 mm at 25°C and 4 mm at 30°C, at 72 h with 12 h photoperiod. Mycelium cottony, with light green spores formed after 96 h, concentrated in the centre of the plate and in a broad concentric ring approximately half-way to the plate edge.

Conidiophores trichoderma-like, pyramidal with opposing branches or isolated, terminating in groups of three to five phialides. Phialides ampulliform to lageniform, constricted below the tip forming a narrow neck, measuring 7.71 ± 1.42 x 2.52 ± 0.32 μm (overall range 5.45–10.75 x 1.89–3.17 μm), base 1.46–2.55 μm (mean 1.99 μm). Conidia globose, subglobose to ovoid 3.90 ± 0.31 x 2.93 ± 0.22 μm (overall range: 3.54–4.65 x 2.55–3.33 μm). Chlamydospores common, terminal and intercalary, typically globose.

Sexual morph: Unknown. Known distribution: Brazil.

#### Other isolates examined

CEN1403, CEN1423. From garlic or onion crop soils.

#### Notes

*Trichoderma azevedoi* is closely related to *Trichoderma rifaii* (a member of the *Trichoderma harzianum* complex). However, *T*. *rifaii* is known only as an endophyte of *Theobroma cacao* and *Theobroma gileri*. *T*. *azevedoi* conidia (mean 3.90 x 2.93 μm) are much larger than *T*. *rifaii* (mean 2.6 x 2.4 μm) and *T*. *azevedoi* produces abundant clamydospores, which have not been reported in *T*. *rifaii*.

**Fig 4:**
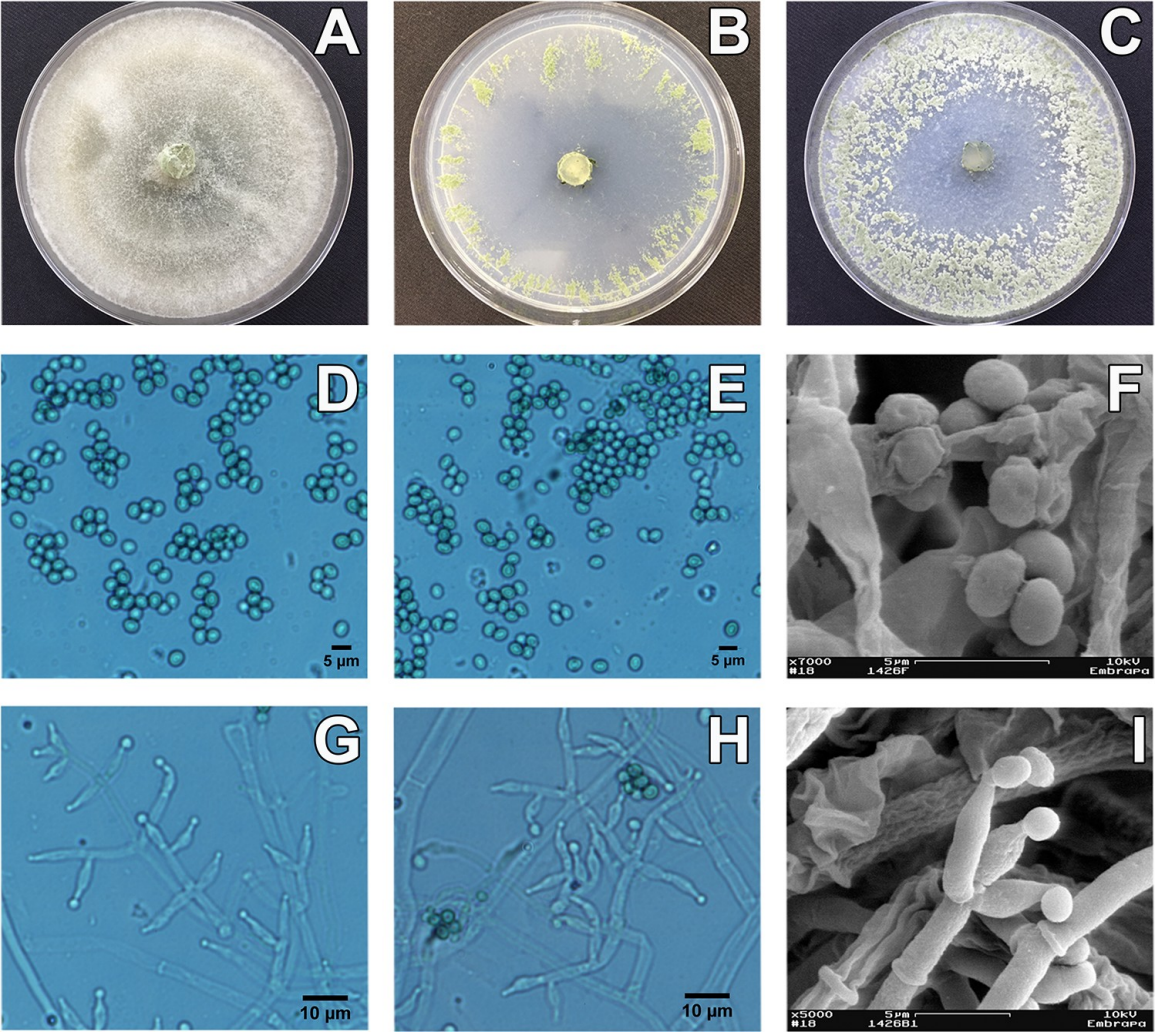
Culture characteristics and morphology of *T*. *peberdyi* sp. nov. strain CEN1426 (holotype). Panels A-I: Growth on three different media, PDA (A); SNA (B); CMD (C); and morphology of the conidia (D, E) and phialides using optical microscopy (G, H) and conidia using electron microscopy (F, I).

***Trichoderma peberdyi*** Valadares-Inglis, M.C. & Inglis, P.W. **sp. nov. Fig 4.**

Mycobank MB830304. [urn:lsid:mycobank.org: 830304]

#### Etmology

Named in honour of John F. Peberdy (Nottingham University, UK), for his important contributions to mycology and fungal biotechnology.

#### Holotype

CEN1426, a freeze dried, metabolically inactive culture deposited in the Herbarium of Embrapa Recursos Genéticos e Biotecnologia (CEN). It was isolated in Itobi—SP state, Brazil, 21^0^ 44^’^ 13” S, 46^0^ 58^’^ 30^”^ E, from onion crop soil, on 02/09/2015, by V. Lourenço Jr & J.B.T. da Silva. An ex-holotype culture of CEN1426 has been deposited in the Embrapa Coleção de Microrganismos para o Controle de Fitopatógenos e Plantas Daninhas, with the accession number BRM46363.

#### Description

On CMD, colony radius 40 mm after 72h at 25 and 30°C with 12 h photoperiod. Colony hyaline in sterile zones with cottony aerial hyphae after 72 h at 25 and 30°C. Spores formed after 120 h at 25°C in pustules concentrated at the plate edge. Light green spores produced at 30°C after 120 h. At 20°C and 12h photoperiod, hyaline mycelium covering entire plate and no spores observed after 120h. No growth at 15 and 35°C. On SNA, colony radius 40 mm after 96 h at 25°C and 30°C, under 12h photoperiod. Colony hyaline with sparse cottony aerial hyphae. Light green spores produced in rays near plate edge after 120 h at 25°C. Sporulation less dense at 30°C. No spores formed at 20°C. No growth observed 15 and 35°C after 120h. On PDA, colony radius 40 mm at 25 and 30°C after 72h under 12h photoperiod. Mycelium cottony, with aerial hyphae covering the entire plate with conidia forming under cottony aerial hyphae after 120h at 25 and 30°C. No diffusible pigments or distinctive odours observed. Conidiophores trichoderma-like, pyramidal with opposing branches or isolated, terminating in groups of two to three phialides. Phialides ampulliform, 7.04 ± 1.01 x 2.67 ± 0.36 (range: 4.91–9.10 x 2.20–3.73 μm), base 1.46–2.55 (mean 1.99 μm). Conidia subglobose to ovoid 3.54–4.65 (3.90) x 2.55–3.33 (2.93) μm, thinning in the proximal region, produced in chains and aggregated in mucilaginous masses. Chlamydospores not observed.

Sexual morph: Unknown. Known distribution: Brazil.

#### Other isolates examined

CEN1387, CEN1388, CEN1389, CEN1390, CEN1391, CEN1392, CEN1393, CEN1398, CEN1457, CEN1458, CEN1425. All from garlic or onion crop soils.

#### Notes

*Trichoderma peberdyi* is closely related to *Trichoderma tomentosum* and *Trichoderma ceraceum*. In comparison with *T*. *tomentosum*, phialides of *T*. *peberdyi* are longer and possess a distinct neck, mostly curved towards the tip. *T*. *peberdyi* conidia are a much lighter green than *T*. *tomentosum* on SNA media and not produced in distinct concentric rings. *T*. *peberdyi* conidia are subglobose to ovoid, larger than *T*. *tomentosum*, a species that produces chlamydospores on CMD, unlike *T*. *peberdyi*. *T*. *peberdyi* is distinct from *T*. *ceraceum* by its lack of diffusible yellow pigment and absence of drops of clear green liquid into which conidia form. *T*. *ceraceum* is known only from the USA.

In trees based on individual markers (S2–S6 Figs), *T*. *peberdyi* sp. nov. was distinct from all other *Trichoderma* species and was well-supported in all but the *act* ML tree. *T*. *azevedoi*, however, appears to be closely related to other neotropical Harzianum clade species, but was clearly distinct and supported in the *act* and *tef1-α* ML trees (S2 and S6 Figs).

### Geographical distribution of *Trichoderma* species in garlic and onion crop soils

The 54 isolates identified to species level, which were collected from eight different sites distributed in four southeastern Brazilian states, fell into three *Trichoderma* sections: *Pachybasium*, *Trichoderma* and *Longibrachiatum* (www.isth.info). The species diversity per collection site varied so that one site yielded a single *Trichoderma* species, two sites yielded two species, one site yielded three species and four sites yielded four species each (Fig 1; Table 1). In terms of crop, garlic (four sites) yielded six *Trichoderma* species and onion crops (six sites) yielded seven different species. Of the three most frequently isolated species in our analysis, *T*. *asperelloides* was isolated from six sites, *T*. *longibrachiatum* was isolated from a single site and one of the new species, *T*. *peberdyi*, was isolated from four sites (Fig 1).

Inferences on local species diversity are tentative at best, however, and would require a much larger quantitative study, possibly using a meta-barcoding approach (reviewed in Kredics et al., 2018). In a study on the diversity of *Trichoderma* species in the Colombian Amazon region, DNA barcoding of 107 strains using ITS and *tef1* sequences showed that three common cosmopolitan species comprise 68% of the studied isolates, with *T*. *harzianum sensu lato* representing 38% of strains, followed by *Trichoderma spirale* at 17% and *T*. *koningiopsis* at 13%, whereas only four putative new taxa were suggested [50]. A larger study of 2078 *Trichoderma* strains collected from agricultural fields in Eastern China, representing four major agricultural provinces, identified 17 known species: *T*. *harzianum* (429 isolates), *T*. *asperellum* (425), *T*. *hamatum* (397), *T*. *virens* (340), *T*. *koningiopsis* (248), *T*. *brevicompactum* (73), *T*. *atroviride* (73), *T*. *fertile* (26), *T*. *longibrachiatum* (22), *T*. *pleuroticola* (16), *T*. *erinaceum* (16), *T*. *oblongisporum* (2), *T*. *polysporum* (2), *T*. *spirale* (2), *T*. *capillare* (2), *T*. *velutinum* (2), and *T*. *saturnisporum* (1) [51]. The authors showed that *Trichoderma* biodiversity in agricultural fields varied by region, crop, and season, where, for example, relative frequencies of *T*. *hamatum* and *T*. *koningiopsis* from rice crop soil were higher than those from wheat and maize soils, suggesting a crop preference of specific *Trichoderma* species. Although this study principally used ITS sequences to identify species and did not split the *T*. *harzianum* species complex along the lines of its currently accepted taxonomic framework [23], there is remarkable overlap with the species recovered in the present study of Brazilian garlic and onion crop soils, despite the large geographical separation. There is accumulating evidence that certain *Trichoderma* species have become highly adapted to agroecosystems. Sixty-five percent of *Trichoderma* species associated with the rhizosphere of maize were shared between samples collected from Austria, Tenerife, Madagascar and New Zealand, whereas *Trichoderma* species associated with endemic plants from the same regions were highly specific and diverse. All analysed rhizosphere samples, however, shared a global *Trichoderma* core community dominated by *T*. *koningii* and *T*. *koningiopsis* [52].

While a comprehensive worldwide survey of the distribution of *Trichoderma* species under the current rapidly evolving taxonomic framework does not yet exist, recent re-evaluations of existing international culture collections and new collecting efforts in under-sampled geographical locations have greatly expanded our knowledge. Pertinent to the new species recovered herein, those most closely related to *T*. *azevedoi* include *T*. *T*. *rifaii*, *T*. *endophyticum* and *T*. *neotropicale*, all of which have been reported to have neotropical distributions [23,33,53]. Species most closely related to *T*. *peberdyi* include *T*. *ceraceum*, first reported from the USA [54] and *T*. *tomentosum*, which is probably cosmopolitan (unpublished Genbank strain data). Among the other *Trichoderma* species recovered in the current study (Table 1), *T*. *lentiforme* has been reported to be neotropical [23], while the remaining species are of worldwide distribution [23,51,55,56].

### Genotypic and phenotypic variability of *Trichoderma* strains

#### AFLP

**Fig 5:**
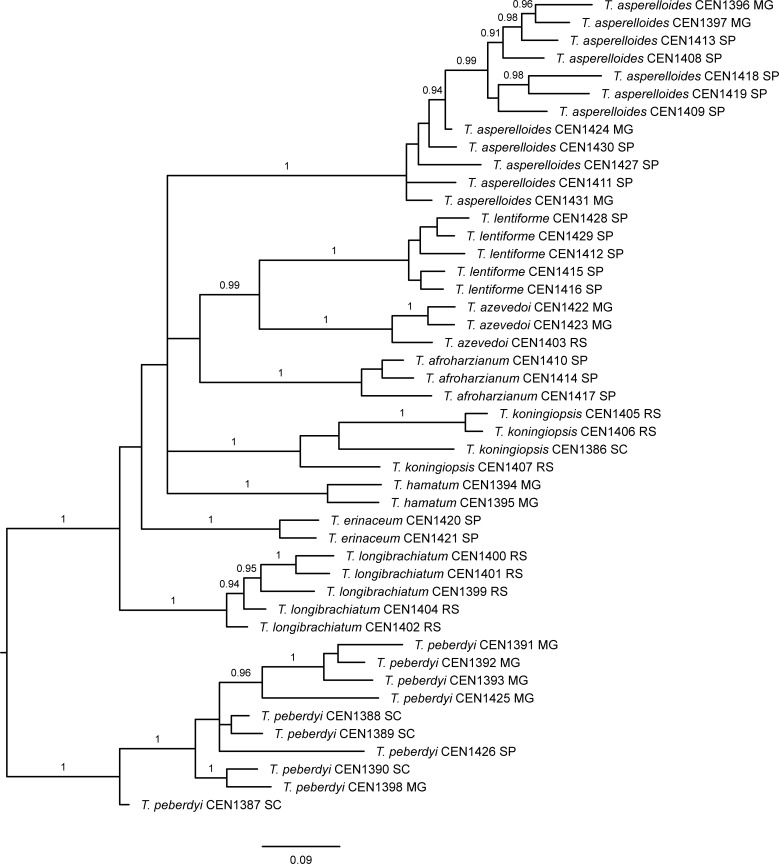
AFLP midpoint rooted Bayesian phylogram. Posterior probabilities are given above branches (>0.9) and the scale bar represents expected changes per site. Species names are followed by strain number and collection location by Brazilian state.

AFLP is a powerful and established molecular tool for the analysis of genetic variation in fungal populations [57]. The combination of six selective AFLP primer pairs (Table 3) yielded 364 binary characters in our selected sample of *Trichoderma* isolates, which were analysed using Bayesian phylogenetic inference (Fig 5). The AFLP clusters agreed closely with the species designations obtained by amplicon sequencing, where each species cluster possessed high posterior support. Closely related species in the Harzianum clade (*T*. *lentiforme*, *T*. *azevedoi*, *T*. *afroharzianum* and *T*. *peberdyi*), were clearly distinguished. However, posterior supports for some of the deeper branches of the tree were poor, as was topological congruence with the DNA sequence-based tree, suggesting that phylogenetic signals were probably saturated at this level or overcome by homoplasy. Modifications of the AFLP protocol, such as the use of *Eco*RI+3 primers for selective PCR, exclusion of smaller fragments or scoring of only major high rfu peaks, might improve phylogenetic resolution in deeper nodes of the resultant tree, but are likely to be at the expense of the ability to discriminate closely-related taxa. Fragment homology has been shown to decrease with greater time since divergence, so that AFLP data are probably best suited for examining phylogeographic patterns within species and among very recently diverged species [58].

In *T*. *asperelloides*, which was one of the most commonly sampled taxa, there was no consistent, well-supported monophyletic grouping of strains according to geographical location (Fig 5). All isolates of this species, however, were collected in the south of MG and the north of SP states, which are contiguous regions (Fig 1) and where populations are possibly not clearly structured. In *T*. *peberdyi* sp. nov., another frequently sampled species, a well-supported clade of four isolates was apparent, all from the municipality of Rio Paranaíba in the mid-west of MG state (Table 1). The clade was further structured so that strain CEN1425, isolated from onion, was differentiated from the other three isolates, which were all isolated from garlic crop soil. The remaining *T*. *peberdyi* strains lacked clear and supported phylogeographic groupings, although most were isolated in the discontiguous SC state, where the outlier strain, CEN1387, was also isolated. Strain CEN1398 from Bueno Brandão-MG grouped with strain CEN1390 from SC. It is unclear if this pattern represents strain dispersal from SC or is the result of limited sampling of a contiguous population of the lineage, since the geographical range of *T*. *peberdyi* is currently unknown. *T*. *peberdyi*, along with our second newly described species, *T*. *azevedoi* sp. nov., possessed the widest geographical range observed in the current study. The two *T*. *azevedoi* strains collected from MG state formed a well-supported clade, distinct from the third strain collected from the distant RS state. The other *Trichoderma* species appeared to be common to only one or a few contiguous locations (Table 1; Fig 1), where mixing and dispersal of haplotypes over shorter distances is probably frequent. Elsewhere, in a study of *Trichoderma* spp. associated with the button mushroom, *Agaricus bisporus*, no clear trend was detected between AFLP clustering and geographic origin of isolated materials [59]. In terms of strain distinction, all of the *Trichoderma* species analysed by AFLP demonstrated significant genetic variability in the Bayesian phylogenetic analysis (Fig 5) and in calculated pairwise genetic distances (Table 5). *T*. *peberdyi* possessed the largest maximum intraspecific genetic distance, although the largest mean intraspecific distance was in *T*. *koningiopsis*.

**Table 5:** Intraspecific genetic distances based on ALFPs.

Species	Mean Distance	Maximum Distance
*T*. *afroharzianum*	0.0659	0.0797
*T*. *asperelloides*	0.0890	0.1209
*T*. *azevedoi*	0.0623	0.0742
*T*. *erinaceum*	0.0549	0.0549
*T*. *hamatum*	0.0769	0.0769
*T*. *koningiopsis*	0.1447	0.1786
*T*. *lentiforme*	0.0500	0.0659
*T*. *longibrachiatum*	0.0703	0.0906
*T*. *peberdyi*	0.1310	0.1978

#### MALDI-TOF

Matrix-assisted laser desorption/ionization mass spectrometry (MALDI-TOF MS) has become an attractive tool for the identification of microorganisms due to short processing time, reliable identification and low per-sample cost. Many filamentous fungi, such as *Aspergillus*, *Fusarium*, *Penicillium* and *Trichoderma*, have been identified by MALDI-TOF [20], where the technique can be used to complement DNA-based identification [60].

**Fig 6:**
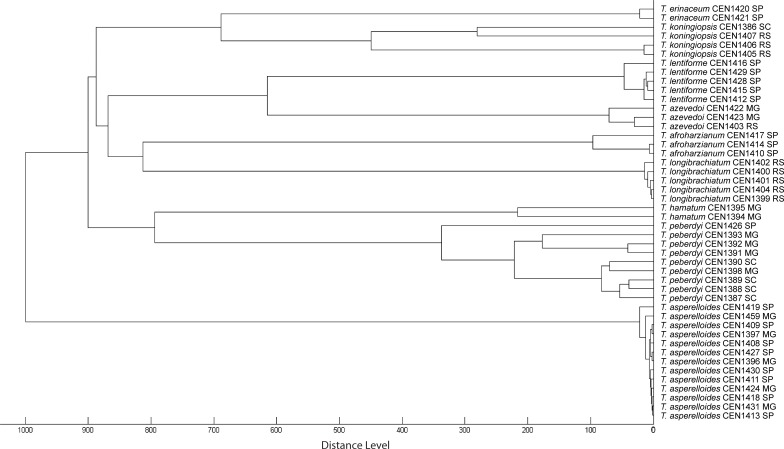
Dendrogram based on MALDI TOF analysis of *Trichoderma* strains isolated from garlic and onion crop soils. Species names are followed by strain number and collection location by Brazilian state.

We selected 46 strains for analysis using MALDI-TOF, representing at least two of each *Trichoderma* species, previously identified by sequence analysis (Fig 2), with the exception of *T*. *asperellum*. Distance-based clustering of MALDI TOF spectra produced a dendrogram (Fig 6) with terminal clusters perfectly matching sequence-based identifications (Fig 2). Echoing the AFLP genotyping result (Fig 5), *T*. *peberdyi* was remarkable for the large phenotypic distance between strains in the MALDI-TOF dendrogram, second only to *T*. *koningiopsis*. In contrast, the *T*. *asperelloides* and *T*. *longibrachiatum* strains, which showed genetic variability in the AFLP analysis, were notably homogenous phenotypically. The most phenotypically diverse species was *T*. *koningiopsis*, which was sister to *T*. *erinaceum*, in agreement with the sequence-based phylogeny (Fig 2B). Otherwise, as was the case with AFLP genotyping, the topology of the MALDI-TOF dendrogram was not congruent with the sequence-based phylogeny, where both typing methodologies appear to be unsuitable for establishing deeper phylogenetic relationships in *Trichoderma*. No exclusive location-correlated groupings were observed in the MALDI-TOF species clusters, although some structure was evident in *T*. *peberdyi*, where a clade containing three strains from SC state was observed, which were joined by a fourth strain from MG (CEN1398). Three other *T*. *peberdyi* strains from MG state formed a sister group and a strain from SP was an outlier in the species. Genetically well-characterized *Trichoderma* species were previously examined using MALDI-TOF, where 129 strains representing 28 species in 8 phylogenetic clades were effectively identified to the species level, providing comparable resolution to ITS sequencing [61]. The authors claimed approximate agreement with the sequence-based phylogeny, which we did not reproduce herein, although this could be due to sampling differences between the two studies.

The major constraint on the use of MALDI-TOF for fungal identification, especially in environmental samples, is the lack of a comprehensive reference spectrum library [62]. Previously, MALDI-TOF was used to identify *Metarhizium* species, where accuracy was progressively improved with the addition of further correctly identified strains to the spectrum library until near perfect matches with DNA-based identifications were obtained [62]. Our analysis was principally directed towards clustering and detection of phenotypic diversity among the onion and garlic-associated strains. However, the technique would appear to be promising for the rapid identification of new *Trichoderma* isolates, since the MALDI-TOF MSP clusters we obtained agreed perfectly with sequence-based identifications. Similarly, MALDI-TOF could be exploited as a fast and economical means of large-scale pre-grouping and triage of anonymous isolates prior to selection of representative individuals for sequence-based phylogenetic identification.

## Conclusions

The large variety of *Trichoderma* species and genotypes identified in a small sample (n = 54) of isolates from garlic and onion crops in South-eastern Brazil represents a considerable resource for the selection of antagonists for biocontrol programs. The biological diversity present is exemplified by the discovery of two new *Trichoderma* species in this sample. While Brazil is among the megadiverse countries, systematic studies on microbial diversity in the range of biomes in the country are currently few [63]. A much larger systematic survey of *Trichoderma* populations associated with both crop and natural soils would enable a clearer picture of the distribution of species in the region. Complimentary sampling of epiphytic and endophytic niches could also broaden the scope for discovery. Such programs also provide the opportunity to preserve distinctive and potentially valuable *Trichoderma* germplasm. Given the laborious nature of pure culture collection and amplicon sequencing for species identification, comprehensive geographical mapping of species could be more efficiently accomplished by metabarcoding, using a sufficiently discriminatory target sequence, such as *tef1-α*.

## Supporting information

S1 Table Genbank accession numbers of reference strains used for phylogenetic analysis. (PDF)

S1 Fig Midpoint rooted maximum likelihood (ML) tree based on the concatenation of *act*, *cal*, ITS, *rpb2* and *tef1-α* matrices. Ultrafast bootstrap values are given above branches (> = 90%) and the scale bar represents expected changes per site. Strains sequenced in the present study are in bold and are followed by CENxxx numbers. Two new *Trichoderma* species, *T*. *azevedoi* and *T*. *peberdyi* are indicated in bold type.
(PDF)

S2 Fig Midpoint rooted maximum likelihood (ML) tree based on the *act* matrix. Ultrafast bootstrap values are given above branches (> = 90%) and the scale bar represents expected changes per site. Strains sequenced in the present study are followed by CENxxx numbers.
(PDF)

S3 Fig Midpoint rooted maximum likelihood (ML) tree based on the *cal* matrix. Ultrafast bootstrap values are given above branches (> = 90%) and the scale bar represents expected changes per site. Strains sequenced in the present study are followed by CENxxx numbers.
(PDF)

S4 Fig Midpoint rooted maximum likelihood (ML) tree based on the ITS matrix. Ultrafast bootstrap values are given above branches (> = 90%) and the scale bar represents expected changes per site. Strains sequenced in the present study are followed by CENxxx numbers.
(PDF)

S5 Fig Midpoint rooted maximum likelihood (ML) tree based on the *rpb2* matrix. Ultrafast bootstrap values are given above branches (> = 90%) and the scale bar represents expected changes per site. Strains sequenced in the present study are followed by CENxxx numbers.
(PDF)

S6 Fig Midpoint rooted maximum likelihood (ML) tree based on the *tef1-α* matrix. Ultrafast bootstrap values are given above branches (> = 90%) and the scale bar represents expected changes per site. Strains sequenced in the present study are followed by CENxxx numbers.
(PDF)

S1 Dataset Concatenated data matrix in Nexus format, containing aligned *act*, *cal*, ITS, *rpb2* and *tef1-α* sequences. (NEX)
