# Supplementary material for: Trichoderma from Brazilian garlic and onion crop soils and description of two new species: Trichoderma azevedoi and Trichoderma peberdyi
Source: PLoS One. 2020 Mar 4;15(3):e0228485. doi: 10.1371/journal.pone.0228485 (PMC7055844; doi:10.1371/journal.pone.0228485)
Supplement: S1 Table — (PDF) [file pone.0228485.s001.pdf]

S1 Table. Genbank accession numbers of reference strains used for phylogenetic analysis.

Species	Strain	Type	ACT	CAL	ITS	RPB2	TEF1
Trichoderma afarasin	Dis 377A		FJ442562	KP115277	FJ442665	FJ442799	FJ463322
Trichoderma afarasin	GJS 99-227	T	FJ442536	FJ442388	AY027784	-	AF348093
Trichoderma afroharzianum	GJS 00-24		FJ442488	AF442880	-	-	-
Trichoderma afroharzianum	GJS 04-186	T	FJ442531	AF442878	FJ442265	FJ442691	FJ463301
Trichoderma aggressivum	DAOM 222156	T	FJ442438	AF442860	NG_064979	FJ442752	AF348098
Trichoderma alni	CBS120.633	T	-	EU498326	EU518651	EU498349	EU498312
Trichoderma amazonicum	IB50	T	KC561140	-	HM142358	HM142367	HM142376
Trichoderma andinense	GJS 09-62		JN133581	JN175414	MH549084	JN175533	JN175587
Trichoderma asperelloides	GJS 04-116		GU248410	-	GU198301	GU248411	GU248412
Trichoderma asperelloides	GJS 04-217		DQ333564	DQ379002	DQ381957	-	DQ381958
Trichoderma asperellum	CGMCC 6422		GU198251	-	KF425754	KF425755	KF425756
Trichoderma asperellum	GJS 91-162		FJ442591	FJ442414	FJ442224	FJ442774	FJ463285
Trichoderma atrobrunneum	GJS 90-254	T	FJ442525	AF442883	NR_137298	FJ442735	FJ463397
Trichoderma atroviride	CBS 142.95	T	DQ111971	DQ122166	MH862505	EU341801	AY376051
Trichoderma austrokoningii	GJS 99-146	T	DQ333551	DQ379003	NR_134364	KJ842161	DQ307561
Trichoderma breve	TC736		-	-	KY687928	KY687984	KY688046
Trichoderma brevicompactum	GJS 04-381		JN133583	JN133522	EU330941	EU338317	AY937453
Trichoderma brunneoviride	CBS 120.928		-	EU498330	EU518661	EU498358	EU498318
Trichoderma camerunense	GJS 99-230	T	FJ442537	AF442875	NR_137300	-	AF348107

Trichoderma capillare	GJS 99-3		-	JN175411	KY225655	JN175529	JN175584
Trichoderma caribbaeum	GJS 98-43		DQ328606	DQ367673	DQ313139	FJ442723.	DQ284976
Trichoderma catoptron	GJS 02-76	T	FJ442584	FJ442387	AY737766	AY391900	AY737726
Trichoderma ceraceum	GJS 95-159		-	-	AF275332	AF545508	AF534603
Trichoderma ceramicum	CBS 114.576	T	FJ442585	FJ442401	AY737764	AF545510	AF534593
Trichoderma cerinum	ATCC MYA-4840	T	-	-	NR_111835	KJ842184.	AY937443.
Trichoderma chlorosporum	GJS 98-1		FJ442559	-	AY737762	AY391907	AY737737
Trichoderma cinnamomeum	GJS 97-237	T	FJ442582	JN133524	AY737759	AY391920	KJ871252
Trichoderma citrinoviride	DAOM 172792	T	-	JN175419	EU280098	EU280036	EU280098
Trichoderma citrinoviride	GJS 92-8		KR812110	JN175424	KR812111	JN175544	JN175595
Trichoderma compactum	YMF1.01693	T	-	-	AY941822	KP115276	KF134798
Trichoderma crassum	GJS 01-227		-	-	-	AY481587	JN133572.
Trichoderma dacrymycellum	WU 29044		-	-	FJ860749	FJ860533	-
Trichoderma dingleyae	GJS 02-50	T	DQ367718	DQ370461	DQ333548	KJ665257	DQ284978
Trichoderma dorotheae	GJS 99-202	T	DQ328603	DQ367706	DQ313145	MH874604	-
Trichoderma effusum	DAOM 230007		-	JN182286	DQ083008	KJ665260	AY937419
Trichoderma endophyticum	PP89		KP115270	KP115283	-	KP115283	-
Trichoderma endophyticum	DIS 217A		FJ442292	FJ442299	FJ442243	FJ442765	FJ463319
Trichoderma erinaceum	DAOM 230019		DQ323450	DQ122163	AY570797	EU248603	-
Trichoderma erinaceum	GJS 02-103		KR873095	KR873096	KR873100	KR873099	KR873097
Trichoderma fertile	DAOM 167161		-	-	NR_134336	AF545546	AF534618

Trichoderma gamsii	GJS 04-09		JN133590	JN133529	DQ315459	JN133561	DQ307541
Trichoderma ghanense	GJS 04-323		JN133591	JN175443	NR_120299	JN175563	JN175613
Trichoderma gillesii	GJS 00-72	T	-	JN175409	-	JN175527	JN175583
Trichoderma guizhouense	GJS 06-100		FJ442506	FJ442343	DQ018116	JQ901400	AY937440
Trichoderma hamatum	DIS 338A		FJ442806	-	FJ442657	FJ150776	-
Trichoderma hamatum	GJS 04-325		EU856267	FJ442286	EU856293	FJ150783	EU856318
Trichoderma harzianum	CBS 226.95	T	FJ442567	FJ577684	-	AF545549	AF348101
Trichoderma harzianum	GJS 95-43		AF442831	AF442864	-	-	-
Trichoderma inhamatum	CBS 273.78	T	FJ442561	AF442891	MH861134	FJ44272	-
Trichoderma intricatum	GJS 97-88	T	AY376684	DQ367680	NR_134343	EU241505	AY376060
Trichoderma konilangbra	CPK 132	T	-	JN182285	MH862712	KJ665284	-
Trichoderma koningii	CBS979.70		-	-	AF359262	-	-
Trichoderma koningii	GJS 89-122		AY376678	DQ367693	AY380902	-	AY376045
Trichoderma koningiopsis	GJS 04-379		DQ367719	DQ367702	DQ323439	-	DQ289001
Trichoderma koningiopsis	S359		-	-	KC874894	KJ665285	KJ665546
Trichoderma koningiopsis	DIS 374A		FJ442597	FJ442418	FJ442213	FJ442730	FJ463288
Trichoderma koningiopsis	GJS 91-6		DQ323446	DQ367705	DQ313135	-	DQ307539
Trichoderma lentiforme	DIS 173D		FJ442443	FJ442290	-	FJ442790	FJ851882
Trichoderma lentiforme	DIS 218E		FJ442449	FJ442296	FJ442220	FJ442793	FJ463310
Trichoderma lentiforme	DIS 354A		FJ442473	FJ442317	FJ442229	FJ442734	FJ463339
Trichoderma lentiforme	GJS 00-22		FJ442487	AF442861	-	FJ442687	-

Trichoderma linzhiense	HMAS 248846	T	-	-	NR_154575	KY687985	KY688047
Trichoderma lixii	GJS 97-96		FJ442533	AF442872	AF443920	AF443938	AF443938
Trichoderma longibrachiatum	CBS 816.68	T	JX238491	EU401459	EU401556	DQ087242	AY865640
Trichoderma longibrachiatum	GJS 07-21		-	JN175393	-	JN175513	JN175569
Trichoderma longipile	DAOM 177227	T	JN133592	JN133534	AY865630	AF545550	AF534622
Trichoderma minutisporum	DAOM 167069	T	DQ111977	DQ122173	NR_111192	KJ665314	KJ665612
Trichoderma neokoningii	GJS 04-216		-	-	DQ841734	KJ665318	DQ841718
Trichoderma neotropicale	CBS 130633	T	KP115268	KP115279	MH865818	-	HQ022771
Trichoderma novae-zelandiae	GJS 81-265		JN133595	JN133536	DQ083019	JN133563	AY937448
Trichoderma oblongisporum	DAOM 167085	T	JN133596	-	NR_138437	AF545551	AF534623
Trichoderma orientale	GJS 10-230		JQ238613	JN175403	EU401550	JN175523	JN175579
Trichoderma ovalisporum	Dis 70A		JQ238613	DQ379005	AY380897	FJ442742	AY376037
Trichoderma paraviridescens	CBS 119.321	T	-	-	NR_134367	-	DQ672610
Trichoderma parepimyces	CBS 122.769	T	-	-	MH863234	FJ860562	FJ860664
Trichoderma petersenii	GJS 04-164		DQ333566	DQ367684	DQ323442	FJ442783	DQ289004
Trichoderma piluliferum	CBS 814.68	T	-	-	AF011935	AF545519	AY737747
Trichoderma pinnatum	GJS 02-120		-	JN175396	-	JN175516	JN175572
Trichoderma pleuroti	CBS 124.387	T	JN133599	JN133539	MH863369	HM142372	HM142382
Trichoderma pleuroticola	CBS 124.383	T	JN133598	JN133538	MH863368	HM142371	HM142381
Trichoderma polysporum	CBS 820.68	T	DQ111976	DQ122172	MH859230	DQ087238	AY605810
Trichoderma pseudokoningii	CBS 408.91	T	-	-	MH862258	AF400740	-

Trichoderma pseudokoningii	GJS 81-300		JN133602	JN175415	DQ083025	JN175534	AY937429
Trichoderma pubescens	DAOM 166162	T	DQ111961	DQ122153	NR_077179	AF545552	EU279963
Trichoderma pyramidale	S73	T	-	-	KX632513	KJ665334	KJ665699
Trichoderma reesei	GJS 97-38		-	JN175432	AJ004962	JN175552	JN175603
Trichoderma rifaii	DIS 337F	T	FJ442471	FJ442315	FJ442621	FJ442720	FJ463321
Trichoderma rifaii	DIS 355B		FJ442474	-	FJ442663	-	FJ463324
Trichoderma rogersonii	GJS 90-78		DQ333558	DQ370457	DQ333549	JN133566	AF534586
Trichoderma rossicum	DAOM 230009	T	JN133605	JN133548	HQ342419	DQ087240	JN133575
Trichoderma saturnisporopsis	S19		-	JN175404	JQ685874	JQ685885	JN175580
Trichoderma simmonsii	GJS 91-138		FJ442526	AF442869	MK346240	FJ442757	KJ665721
Trichoderma sinense	DAOM 230004	T	-	JN175410	NR_134425	JN175528	KJ713191
Trichoderma solani	GJS 08-81		-	JN175426	-	JN175546	JN175597
Trichoderma spirale	CBS 120.963		FJ442552	FJ442363	FJ442363	-	FJ463291
Trichoderma spirale	DAOM 183974	T	FJ442818	FJ442395	NR_077177	AF545553	EU280049
Trichoderma stramineum	GJS 02-84	T	FJ442583	FJ442386	NR_134347	AY391945	AY737746
Trichoderma strictipile	DAOM 167072		FJ442586	FJ442399	FJ442219	KJ842182	AY937450
Trichoderma strigosum	DAOM 166121	T	EU856277	JN133544	DQ083027	AF545556	EU280019
Trichoderma stromaticum	GJS 00-02		FJ442603	HQ342333	FJ442675	FJ442714	FJ463361
Trichoderma taiwanense	GJS 95-93	T	DQ323455	DQ367685	DQ313141	JN715608	DQ284973
Trichoderma tawa	GJS 97-174	T	FJ442570	FJ442406	AY737756	KJ842187	FJ463313
Trichoderma theobromicola	DIS 85F	T	DQ111955	DQ122148	DQ109525	FJ150786	DQ109539

Trichoderma tomentosum	DAOM 178713A	T	JN133612	-	NR_134357	AF545557	EU279969
Trichoderma tomentosum	CBS 120.637		-	-	FJ860744	FJ860532	FJ860629
Trichoderma tomentosum	TRS82		-	-	KP009287	KP009177	KP008914
Trichoderma velutinum	DAOM 230013		JN133614	JN133550	-	JN133569	AY605803
Trichoderma virens	GJS 01-287		-	FJ442404	DQ083023	EU341804	AY750894
Trichoderma viride	CBS 119.325	T	-	-	NR_138441	EU711362	DQ672615
Trichoderma viride	GJS 90-79		DQ333563	DQ367707	DQ381956	-	DQ381959
Trichoderma viridescens	CBS 433.34	T	-	-	MH855595	-	AF456905
