# Supplementary figures and images for: Trichoderma from Brazilian garlic and onion crop soils and description of two new species: Trichoderma azevedoi and Trichoderma peberdyi
Source: PLoS One. 2020 Mar 4;15(3):e0228485. doi: 10.1371/journal.pone.0228485 (PMC7055844; doi:10.1371/journal.pone.0228485)

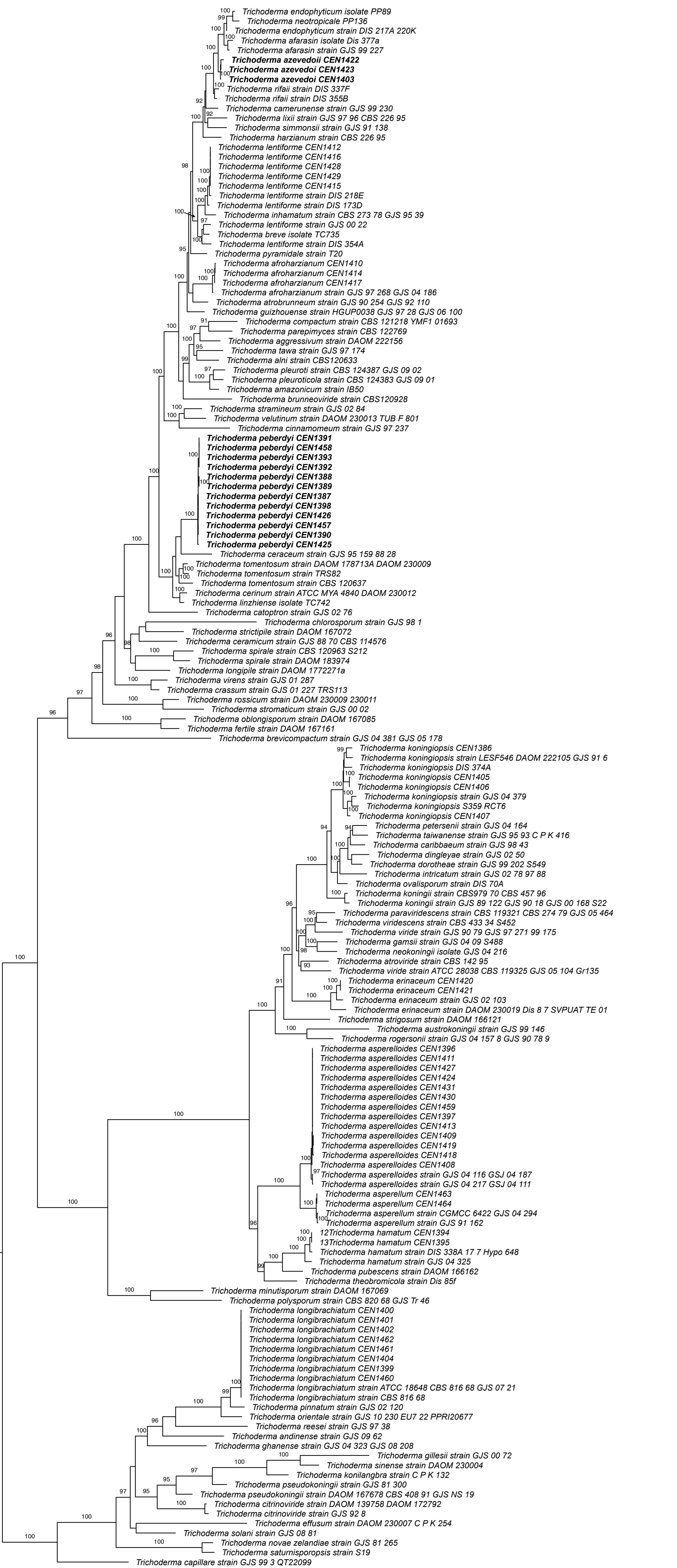

0.07

Supplement: S1 Fig — Ultrafast bootstrap values are given above branches (> = 90%) and the scale bar represents expected changes per site. Strains sequenced in the present study are in bold and are followed by CENxxx numbers. Two new Trichoderma species, T. azevedoi and T. peberdyi are indicated in bold type. (PDF) [file pone.0228485.s002.pdf]

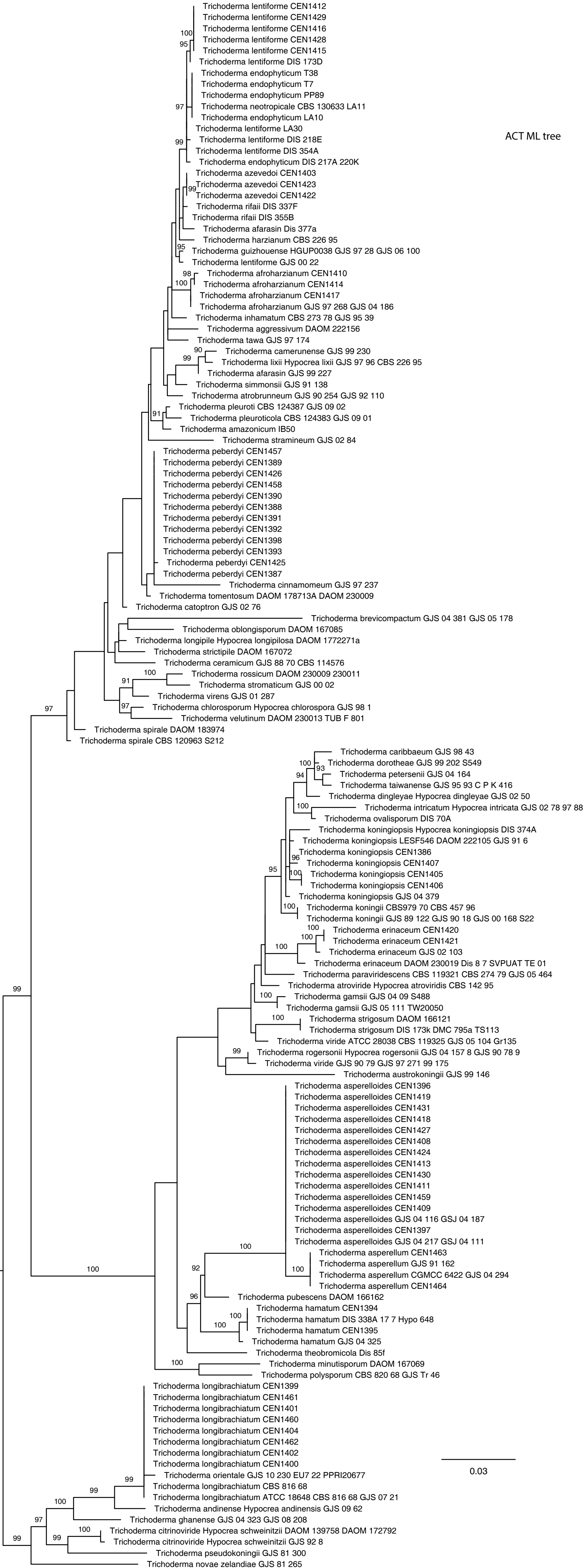

Supplement: S2 Fig — Ultrafast bootstrap values are given above branches (> = 90%) and the scale bar represents expected changes per site. Strains sequenced in the present study are followed by CENxxx numbers. (PDF) [file pone.0228485.s003.pdf]

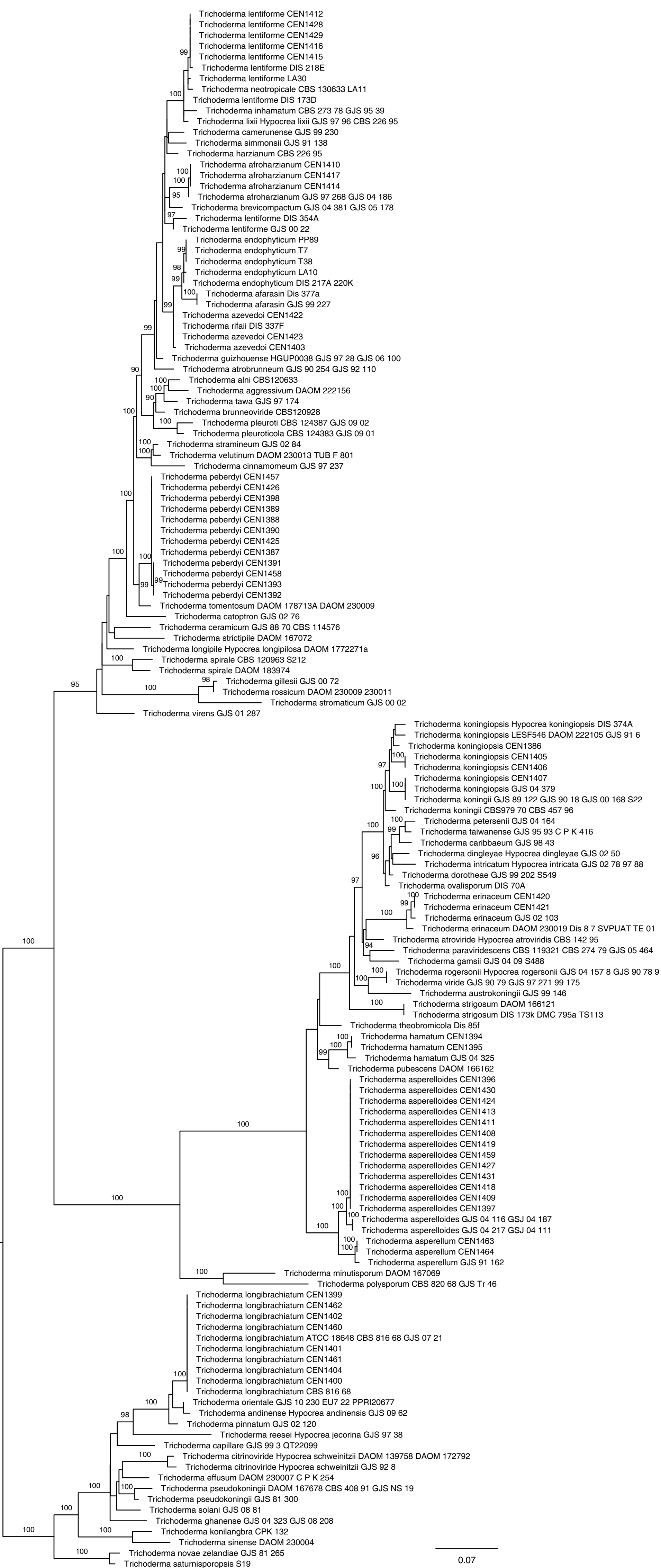

0.07

Supplement: S3 Fig — Ultrafast bootstrap values are given above branches (> = 90%) and the scale bar represents expected changes per site. Strains sequenced in the present study are followed by CENxxx numbers. (PDF) [file pone.0228485.s004.pdf]

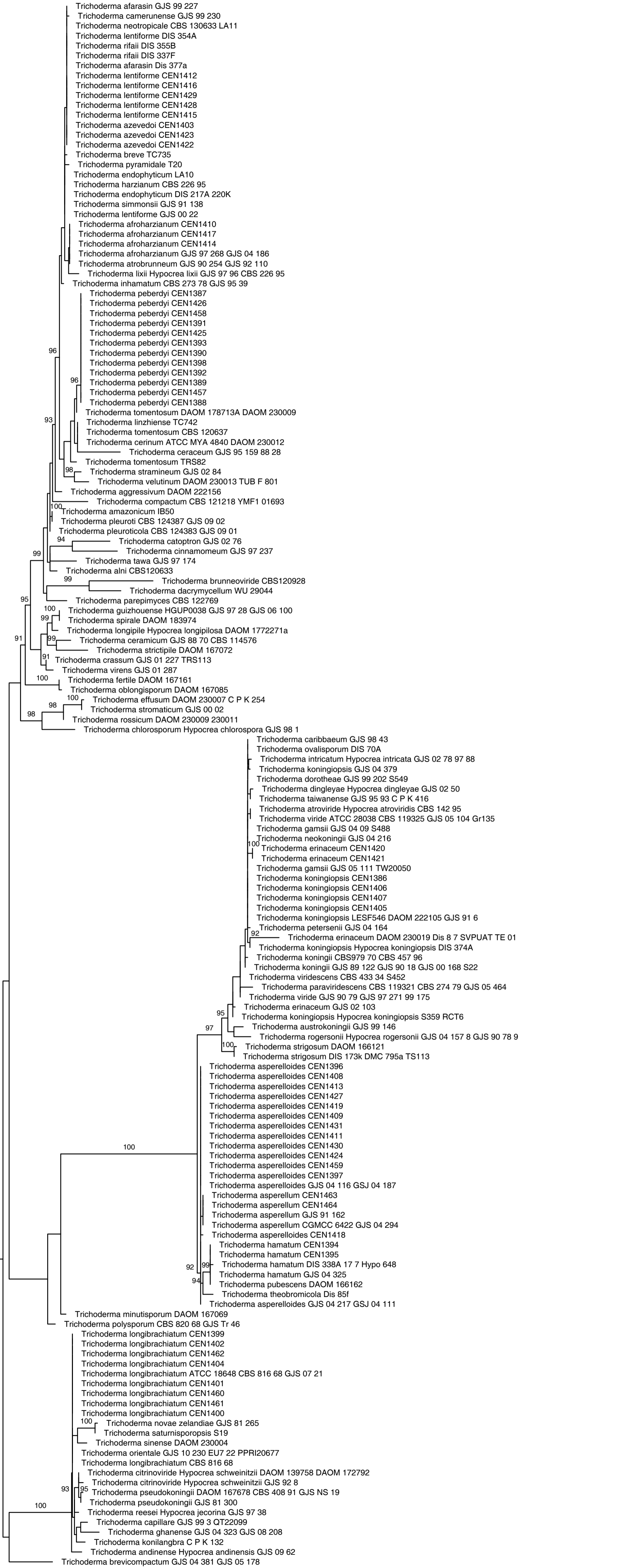

0.04

Supplement: S4 Fig — Ultrafast bootstrap values are given above branches (> = 90%) and the scale bar represents expected changes per site. Strains sequenced in the present study are followed by CENxxx numbers. (PDF) [file pone.0228485.s005.pdf]

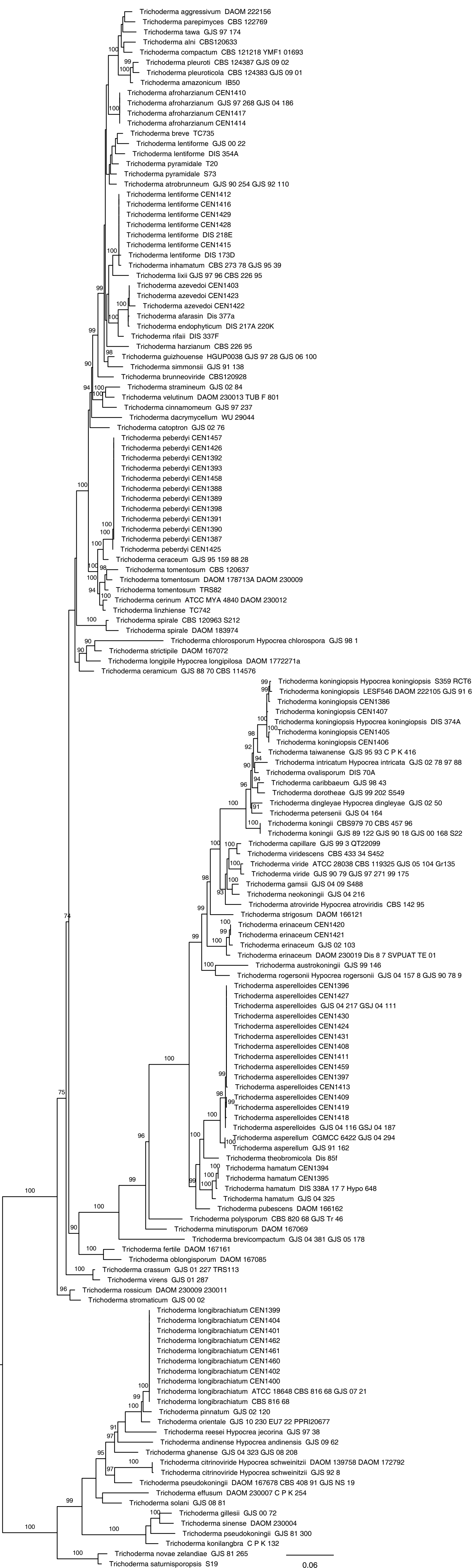

0.06

Supplement: S5 Fig — Ultrafast bootstrap values are given above branches (> = 90%) and the scale bar represents expected changes per site. Strains sequenced in the present study are followed by CENxxx numbers. (PDF) [file pone.0228485.s006.pdf]

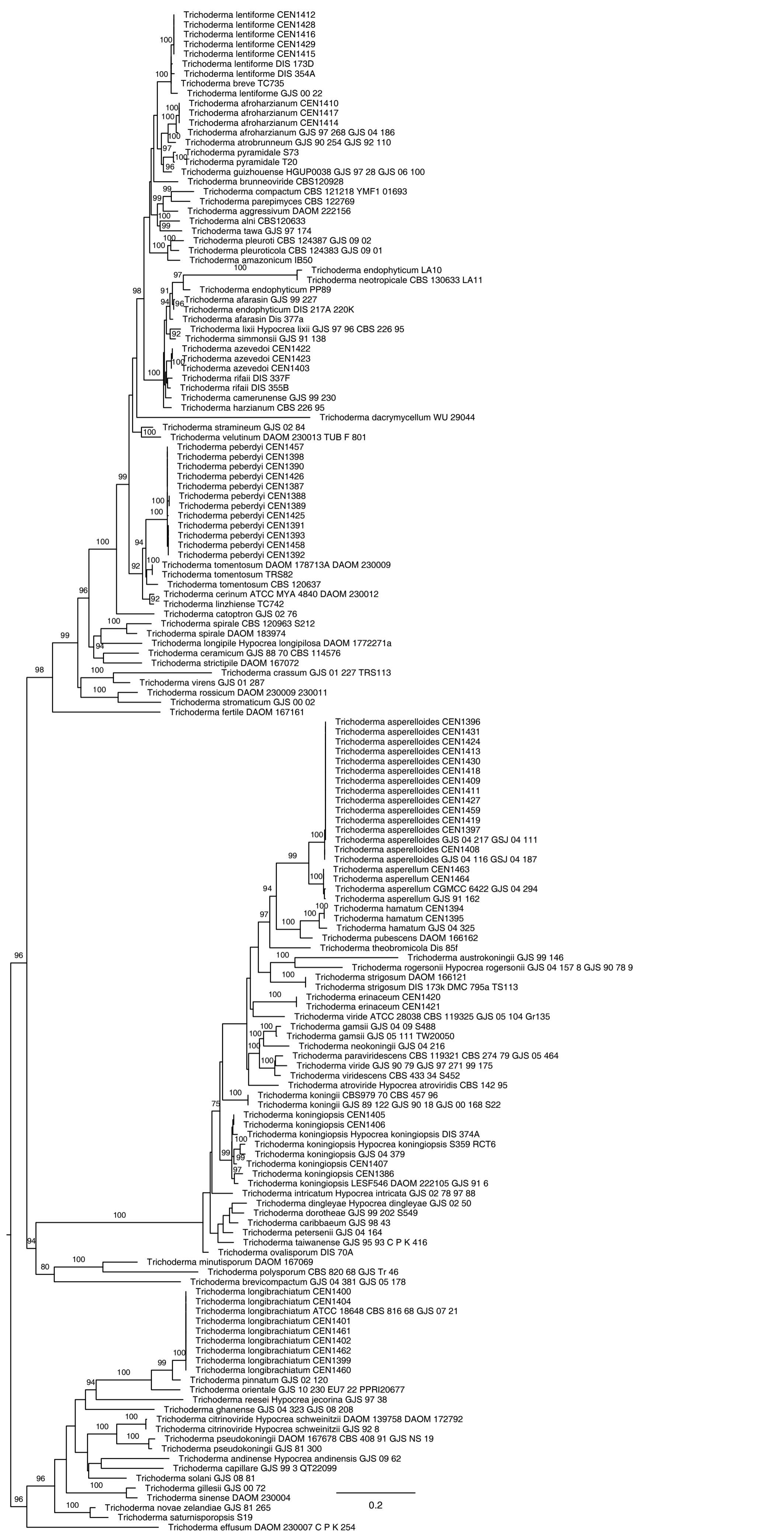

Supplement: S6 Fig — Ultrafast bootstrap values are given above branches (> = 90%) and the scale bar represents expected changes per site. Strains sequenced in the present study are followed by CENxxx numbers. (PDF) [file pone.0228485.s007.pdf]
